# Environmental carcinogens disproportionally mutate genes implicated in neurodevelopmental disorders

**DOI:** 10.3389/fnins.2023.1106573

**Published:** 2023-08-03

**Authors:** Brennan H. Baker, Shaoyi Zhang, Jeremy M. Simon, Sarah M. McLarnan, Wendy K. Chung, Brandon L. Pearson

**Affiliations:** ^1^Department of Environmental Health Sciences, Mailman School of Public Health, Columbia University, New York, NY, United States; ^2^Master of Public Health Program, Department of Epidemiology, Mailman School of Public Health, Columbia University, New York, NY, United States; ^3^Department of Genetics and Neuroscience Center, University of North Carolina at Chapel Hill, Chapel Hill, NC, United States; ^4^Department of Pediatrics and Medicine, Columbia University Irving Medical Center, New York, NY, United States

**Keywords:** somatic mutation, mutagenesis, *de novo* mutation, carcinogen, neurodevelopmental disorders, autism

## Abstract

**Introduction:**

*De novo* mutations contribute to a large proportion of sporadic psychiatric and developmental disorders, yet the potential role of environmental carcinogens as drivers of causal *de novo* mutations in neurodevelopmental disorders is poorly studied.

**Methods:**

To explore environmental mutation vulnerability of disease-associated gene sets, we analyzed publicly available whole genome sequencing datasets of mutations in human induced pluripotent stem cell clonal lines exposed to 12 classes of environmental carcinogens, and human lung cancers from individuals living in highly polluted regions. We compared observed rates of exposure-induced mutations in disease-related gene sets with the expected rates of mutations based on control genes randomly sampled from the genome using exact binomial tests. To explore the role of sequence characteristics in mutation vulnerability, we modeled the effects of sequence length, gene expression, and percent GC content on mutation rates of entire genes and gene coding sequences using multivariate Quasi-Poisson regressions.

**Results:**

We demonstrate that several mutagens, including radiation and polycyclic aromatic hydrocarbons, disproportionately mutate genes related to neurodevelopmental disorders including autism spectrum disorders, schizophrenia, and attention deficit hyperactivity disorder. Other disease genes including amyotrophic lateral sclerosis, Alzheimer’s disease, congenital heart disease, orofacial clefts, and coronary artery disease were generally not mutated more than expected. Longer sequence length was more strongly associated with elevated mutations in entire genes compared with mutations in coding sequences. Increased expression was associated with decreased coding sequence mutation rate, but not with the mutability of entire genes. Increased GC content was associated with increased coding sequence mutation rates but decreased mutation rates in entire genes.

**Discussion:**

Our findings support the possibility that neurodevelopmental disorder genetic etiology is partially driven by a contribution of environment-induced germ line and somatic mutations.

## Introduction

While cancer epidemiologic studies have a long history of integrating genetic and environmental factors into disease causation (Shields and Harris, 2000), researchers, with small exception (Kinney et al., 2010; Pugsley et al., 2021), have not readily implicated environmentally-induced mutations as etiological drivers of neurodevelopmental disorders (NDD) and other diseases. *De novo* mutations contribute to a large proportion of sporadic cases of ASD, schizophrenia, and intellectual disability (De Ligt et al., 2012; Xu et al., 2012; Fromer et al., 2014; Iossifov et al., 2014), yet the underlying mutational processes have not been interrogated, or have been attributed to intrinsic mutational processes (e.g., random replication error) rather than environmental carcinogens. Similarly, environmental exposures may be responsible for a large proportion of NDD (Landrigan, 2010; Bellinger, 2012; Rauh and Margolis, 2016). While potential underlying molecular mechanisms such as epigenetics have been explored in great detail (Perera and Herbstman, 2011; Tran and Miyake, 2017; Emberti Gialloreti et al., 2019), environmentally induced mutation remains a strong yet generally untested candidate mechanism that may link environmental exposures to neurodevelopment (Kinney et al., 2010; Pugsley et al., 2021). For instance, PAHs—a class of chemicals found in tobacco smoke and air pollution—form metabolites in the body that bind with DNA and promote mutation (Whyatt et al., 1998). Consequently, PAHs are well known causes of cancer (Boffetta et al., 1997; Kriek et al., 1998; Kim et al., 2013). Epidemiologic studies have linked prenatal PAH exposure to cognitive developmental delays, reduced intelligence, and ASD (Perera et al., 2006; Edwards et al., 2010; von Ehrenstein et al., 2014; Jedrychowski et al., 2015). However, no studies have examined whether mutations in NDD genes induced by PAHs and other environmental exposures contribute to these epidemiologic associations despite evidence that NDD genes are generally longer (King et al., 2013; Sugino et al., 2014; Gabel et al., 2015) and show considerable overlap with cancer driver genes (Crawley et al., 2016; Qi et al., 2016). To test the hypothesis that NDD genes are more susceptible to mutagens than non-NDD genes, we analyzed a whole genome sequencing (WGS) dataset containing nearly 200,000 single nucleotide substitution mutations in human induced pluripotent stem cell (iPSC) clonal lines exposed to 12 classes of environmental carcinogens (Kucab et al., 2019). We assessed the susceptibility to environmental mutation of genes and disease-associated gene sets by (1) evaluating gene ontology for top mutated genes; (2) developing an online tool for assessing the propensity of 12 mutagen classes to cause mutations in gene sets associated with specific human diseases; (3) investigating gene length, expression, and GC content as potential drivers of elevated mutability using Quasi-Poisson models; and (4) testing whether specific disease-related genes are enriched for bulky DNA adduct repair.

## Materials and methods

### Environmental mutation vulnerability of disease genes

Analyses were performed using R (Team, 2018). We analyzed the substitution mutations from 324 iPSC subclones dosed with 79 environmental carcinogens (Kucab et al., 2019). From whole-genome-sequencing data at ~ 30-fold depth, Kucab et al. (2019) called mutations in subclones subtracting on the primary iPSC parental clone. We compared the observed rates of exposure-induced mutations in disease-related gene sets with the expected rates of mutations based on control genes randomly sampled from the genome. Disease gene sets contained 91 ASD (Abrahams et al., 2013), 104 schizophrenia (Wang et al., 2019), 25 ADHD (Demontis et al., 2019), 33 Alzheimer’s (Giri et al., 2016), 18 ALS (Association T.A., 2019), 81 type 2 diabetes (Mahajan et al., 2014), 80 coronary artery disease (Nikpay et al., 2015), 96 obesity (Locke et al., 2015), 253 congenital heart disease (Jin et al., 2017), and 31 orofacial cleft genes (Beaty et al., 2016; Supplementary Table S1). Gene sets were either curated (i.e., published in review articles or curated by scientific organizations) or based on genes with significant disease-associated loci from genome wide association studies (GWAS). We included adult onset, congenital, heritable, and life-style-associated diseases to determine if our hypothesized NDD enrichment was specific. Since our analyses were restricted to just a handful of disease gene sets and results could depend on the methods of gene set curation, we created an online tool where custom gene lists can be queried using the algorithm we generated.^1^ Using this tool, users may input more up-to-date gene lists. For example, our ASD list included all genes labeled as high confidence by the Simons Foundation Autism Research Initiative (SFARI) at the time of the analysis, but SFARI is constantly updating this gene list as our understanding of the genetic basis of ASD evolves.

To determine expected mutation rates, we randomly sampled 1,000 sets of 300 genes from the human genome and used the iPSC mutation dataset (Kucab et al., 2019) to calculate average rates of mutation per-gene-per-treated iPSC subclone within each exposure class. Our unit of analysis was mutations per gene, so it was not necessary to match the number of randomly sampled genes with the number of genes in each disease set. To check this assumption, we plotted the relationship between the size of randomly sampled gene sets, varying from 10 to 300 genes, with the number of mutations per subclone treated with the radiation class of chemicals. To characterize the degree to which certain disease gene sets were mutated more than expected, we compared these hypothesized expected mutation rates to the mutation rates for each disease gene set within each environmental exposure in clonal iPSC cultures (Kucab et al., 2019) using two-sided exact binomial tests. For a given chemical exposure and disease gene set, the exact binomial test null hypothesis was that the disease gene set had the same per gene mutation rate as the per gene mutation rate of the 1,000 sets of 300 genes described above. Rejection of the null hypothesis indicated that the disease gene set was mutated more or less than the mutation rate of randomly sampled genes. Single genes were allowed to contribute multiple mutations to the mutation rate numerators. Significance was assessed at alpha level 0.05 with table wide Bonferroni corrections. This analysis was repeated for mutations in entire genes as well as coding sequence (CDS) mutations determined using the Ensembl variant effect predictor (McLaren et al., 2016). Although entire genes contain introns and other non-coding sequence, a large proportion of GWAS signals map to non-coding regions (Zhang and Lupski, 2015), so variants in these loci may still contribute to disease.

For mutations in entire genes in PAH-treated iPSCs, we conducted a sensitivity analysis by calculating *p*-values from empirical null distributions rather than from exact binomial tests. Monte Carlo null distributions for each disease gene set were obtained by randomly sampling 1,000 sets of genes from the human genome equal to the number of genes in a given disease gene set. The total number of mutations in each randomly sampled gene set was determined. Two-tailed p-values were calculated as the proportion of randomly sampled gene sets mutated more or less than the comparison disease gene set, whichever was smallest, multiplied by two.

To externally-validate this approach, we repeated the gene mutation analysis in an independent dataset of human WGS data from 14 lung cancers from individuals living in highly polluted regions (Yu et al., 2015). Because PAHs are a major component of pollution, we hypothesized that mutational patterns would be similar between these samples and the PAH-treated iPSCs.

We conducted a sensitivity analysis to explore the role of gene length in environmental mutagen vulnerability. In this analysis, a selected NDD gene list was created by combining all ASD, ADHD, and schizophrenia genes from the lists described above. We then divided the list into four separate lists based on gene length quartiles, and repeated the above analysis for mutations in entire gene bodies.

In an additional sensitivity analysis, we explored the mutational susceptibility of cancer driver genes, and genes with overlap between cancer and NDD. We utilized a list of 233 high confidence cancer genes with confidence scores ≥ 1.5 based on a scoring system developed by (Bailey et al., 2018), and a list of 14 genes that overlap between this cancer gene list and the selected NDD gene list described above.

### Gene ontology

We performed gene ontology (GO) analysis on all genes which contained coding sequence (CDS) variants in PAH-treated iPSCs. GO analysis was performed using FUMA with ensembl version 92, protein coding genes set as the background, and a Bonferroni correction (Watanabe et al., 2017). In an additional sensitivity analysis, we included all genes with CDS mutations in iPSCs exposed to all environmental mutagens rather than just PAH-treated iPSCs.

### Sequence characteristics and mutation vulnerability

Autism spectrum disorder-implicated NDD genes tend to be longer than other genes (King et al., 2013; Zylka et al., 2015). To visually examine if vulnerability of neurodevelopmental genes or CDS to mutagens is attributable to gene length, we plotted the distributions of entire gene and CDS lengths for our disease gene sets, along with the distributions of lengths for entire genes and CDS mutated entirely at random. Random mutations were modeled by randomly sampling (i.e., mutating) 100,000 nucleotides from all genes or all CDS in the human genome, so the probability of a sequence being mutated was entirely governed by its length.

To further explore associations of length with mutability, we modeled the effects of sequence length, expression, and percent GC content on mutation rate using multivariate Quasi-Poisson regressions, with separate models for mutations in CDS and entire genes. Gene and CDS start and stop positions were obtained from GENCODE Release 38^2^ and used in conjunction with the “BSgenome.Hsapiens.UCSC.hg38” R package to calculate genomic sequence lengths (end minus start position) and GC content (proportion of sequence positions with either a G or C nucleotide). When modeling associations of sequence properties with CDS mutations, CDS lengths and GC content were computed per gene: all CDS segments within a single gene were summed as the total coding sequence length, and CDS GC content was calculated per gene rather than per individual CDS segment. Gene expression data were reads per kilobase of transcript per million mapped reads (RPKM) obtained from RNA-seq of iPSCs generated using the Sendai virus method (Churko et al., 2017), the same method used to create the iPSCs used by Kucab et al. (2019). After excluding genes with missing length, expression, or GC content data, Quasi-Poisson models included 75,756 gene and 1,852 CDS mutations. Coefficients from these models were multiplied by the interquartile range (IQR) for each variable and then exponentiated into rate ratios per IQR increase.

### Local sequence and mutation vulnerability

To explore the role of local sequence context on mutability, we aligned 7-mers centered on each gene or CDS mutation identified by Kucab et al. (2019), along with 50,000 7-mers randomly sampled from the human genome. We performed this analysis for all mutations, and stratified by chemical exposure class. We also examined the role of local sequence context by generating COSMIC signatures (Tate et al., 2019) for *de novo* mutations in individuals with neuropsychiatric diseases, including 42,607 ASD cases (Feliciano et al., 2018; Zhou et al., 2021), 617 schizophrenia cases (Fromer et al., 2014), and 145 individuals with severe intellectual disability (De Ligt et al., 2012; Rauch et al., 2012).

### Polycyclic aromatic hydrocarbon adduct repair associated mutation

To determine if NDD genes are linked to PAH adduct repair, we analyzed an existing genome-wide PAH adduct repair assay dataset (Li et al., 2017) to see if adducts are preferentially located in specific disease-related gene sets. Genome-wide PAH adduct repair data come from translesion excision repair-sequencing (tXR-seq) of GM12878 cells, which were grown to ∼ 80% confluence before treatment with 2 μM benzo[a]pyrene diol epoxide-deoxyguanosine for 1 h at 37°C in a 5% CO_2_ humidified chamber. tXR-seq captures all DNA damage, regardless of whether or not it is repaired (Li et al., 2017). We computed the average DNA damage enrichment across each gene or CDS in all 10 disease-related gene sets by inputting bigWig files from Li et al. (2017) into the deepTools2 ‘computeMatrix’ function (Ramírez et al., 2016). By default, the ‘computeMatrix’ function scales input sequences to the same length. Output enrichment values from the ‘computeMatrix’ function are based on the units of the input bigWig files, which, in this case, were tXR-seq counts normalized for total sequencing depth on each chromosome (Li et al., 2017). We used one-way ANOVA to compare levels of DNA damage in genes, which were normally distributed, between disease gene sets, and performed pairwise contrasts with a false discovery rate correction. Kruskal–Wallis and Dunn’s Test were employed for CDS DNA damage data, which were not normally distributed.

## Results

### Environmental mutation vulnerability of disease genes

**Figure 1:**
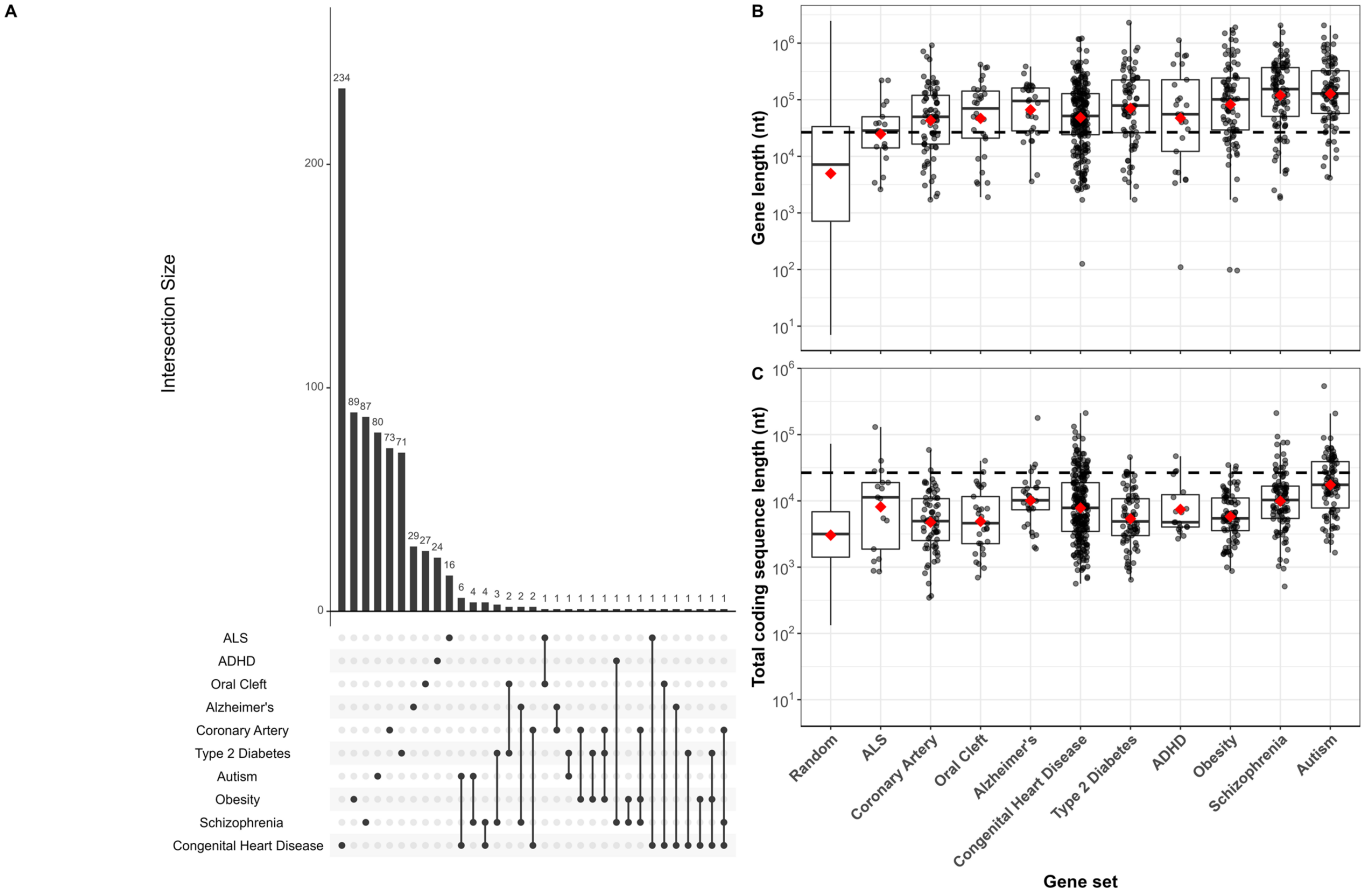
**(A)** Gene set intersection plot. Horizontal bars depict gene set sizes. Gene sets included in each intersection are indicated by a single or connected dot(s), and sizes of the intersections are indicated by vertical bars. For single, unconnected dots, vertical bars indicate the number of genes exclusive to that set. For example, among 253 Congenital Heart Disease genes, 6 overlapped with Autism genes, while 234 had no overlap with other gene sets. Intersections with zero overlap not shown. **(B,C)** Sequence length boxplots with median and 1.5 interquartile range (IQR) whiskers shown for each disease implicated gene set for entire genes **(B)** and coding sequence **(C)**. Red diamonds indicate mean sequence length for each set indicated on x-axis, and dashed black line across entire plot indicates mean gene length of randomly mutated genes (modeled by randomly sampling (i.e., mutating) 100,000 nucleotides from all genes or all CDS in the human genome). Gene and CDS boxplots also shown for all 300,000 randomly sampled sequences (1,000 sets of 300 genes or CDS).

There were 769 unique genes across all ten disease gene sets, and overlap between sets was minimal (Figure 1A). Among the 183,133 substitution mutations identified by Kucab et al. (2019), 92,204 occurred in known genes. Across all chemical treatments, all disease gene set genes had a combined 7,587 total mutations. Per-nucleotide mutation rates for each chemical by disease gene set combination ranged from 0 to 5.77 × 10^−7^. We plotted the distributions of entire gene and CDS lengths for our disease gene sets, along with the distributions of lengths for the 1,000 sets of 300 randomly sampled gene and CDS sequences (Figures 1B,C). NDD and metabolic disease gene sets such as ASD, schizophrenia, ADHD, obesity, and type-2 diabetes contained the longest genes, while ALS genes and randomly sampled genes were the shortest (Figure 1B). On the other hand, average CDS lengths for most disease gene sets were comparable (Figure 1C).

**Figure 2:**
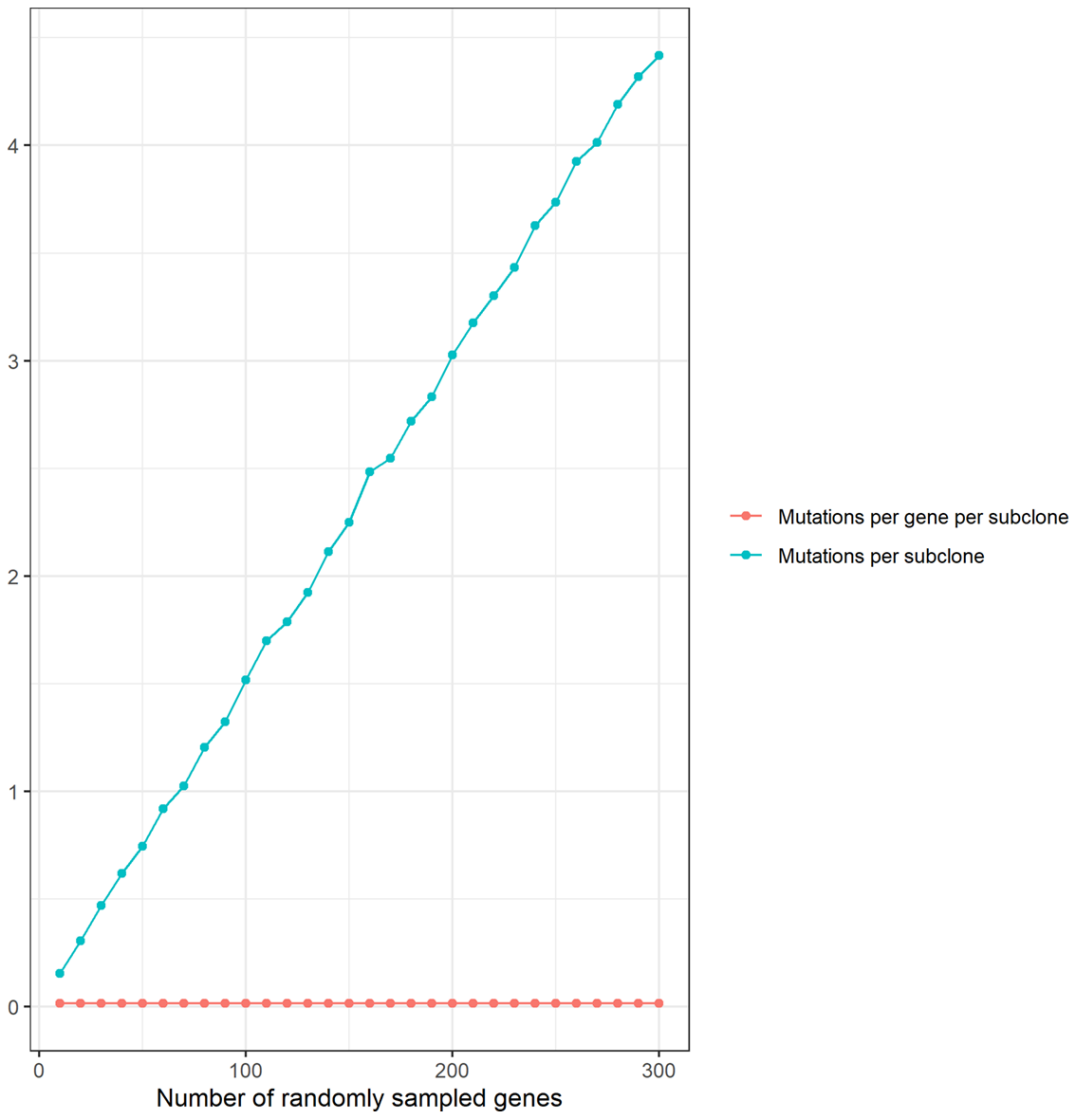
Thousand sets of genes were randomly sampled for each gene set size ranging from 10 to 300 in intervals of 10. For each random gene set size, the number of mutations in subclones treated with the radiation class of chemicals was determined. Mean mutations per subclone and mutations per gene per subclone were plotted against the random set size.

We compared per gene mutation rates in our disease gene sets with genes randomly sampled from the human genome (1,000 sets of 300 genes). Mutations per subclone increased linearly with the number of randomly sampled genes while the number of mutations per gene per subclone remained flat (Figure 2), confirming that it was not necessary to match the number of randomly sampled genes with the number of genes in each disease set.

**Figure 3:**
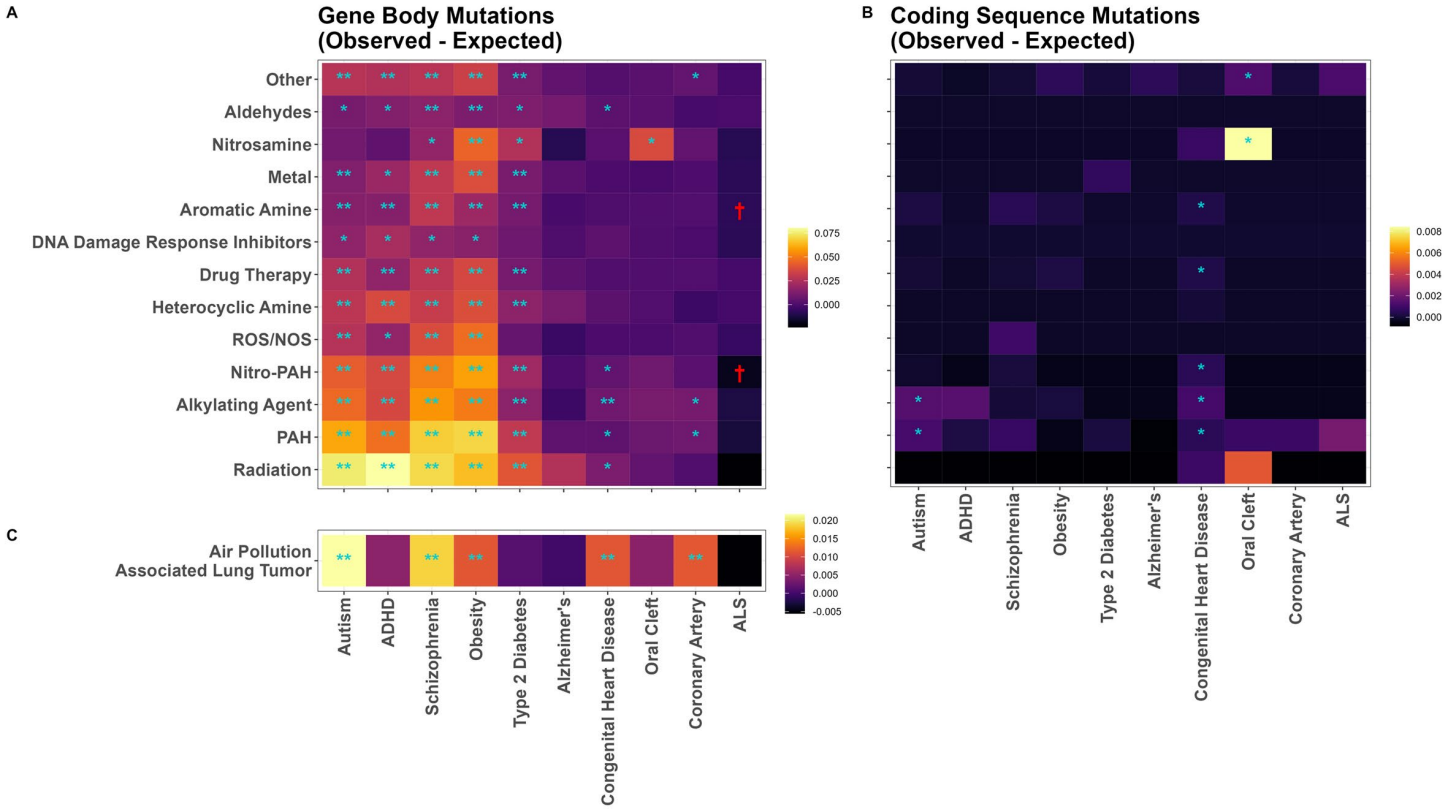
Mutation vulnerability of disease associated genes to various carcinogens. Heatmaps of observed minus expected mutations in disease gene sets for **(A)** mutations in gene body sequence following chemical treatment in iPSC, **(B)** mutations in coding sequences following chemical treatment in iPSC, and **(C)** mutations in 14 human lung cancers from individuals living in highly polluted regions. Significant levels from exact binomial tests. Higher mutation rates: ^*^p < 0.05; ^**^Bonferroni-adjusted *p* < 0.05. Lower mutation rates: ^†^*p* < 0.05.

The most mutagenic exposures were radiation and PAHs, which induced an average of 0.066 and 0.058 substitution mutations per-gene-per-treated iPSC subclone, respectively across our 10 disease related gene sets (Figure 3A; Supplementary Table S2). ASD, ADHD, schizophrenia, obesity, and type-2 diabetes genes were mutated significantly more than expected by almost every chemical class. Congenital heart disease, oral cleft, and coronary artery disease were rarely mutated more than expected; while Alzheimer’s disease genes were never mutated more than expected (Figure 3A; Supplementary Table S3). There was some evidence for ALS genes being mutated less than the expected per-gene mutation rate by aromatic amines and nitro-PAHs, although these differences were not statistically significant after Bonferroni correction. Results were similar in a sensitivity analysis calculating *p*-values from Monte Carlo empirical null distributions for PAH-treatment mutations (Supplementary Figure S1). As in the main analysis, ASD, ADHD, schizophrenia, obesity, and type-2 diabetes genes were mutated significantly more than expected.

Among the 2,061 identified coding sequence variants, these overarching patterns were not observed (Figure 3B). While there was evidence for exposure causing more coding sequence mutations than expected for 9 specific exposure/disease combinations, none of them remained statistically significant following Bonferroni correction (Figure 3B).

We repeated this analysis in an independent dataset of human WGS data from 14 lung cancers from individuals living in highly polluted regions. In these samples, the greatest increases in observed over expected gene mutations were in genes related to ASD, schizophrenia, and obesity (Figure 3C). However, contrasting with the mutational patterns in PAH-exposed iPSCs, ADHD genes were not mutated more than expected, and genes associated with congenital heart defects were mutated more than expected even after Bonferroni correction (Figure 3B).

Average gene lengths of disease gene sets were consistent with patterns of mutability. For instance, ASD, schizophrenia, obesity, and ADHD genes, which were on average longer than other disease-related genes, had the greatest increases in observed versus expected chemical-induced mutations, while ALS, coronary artery disease, oral cleft, and Alzheimer’s disease genes, which are much shorter in length, were not mutated more than expected (*cf.* Figures 1B, 3A). Genes mutated entirely at random (modeled by randomly sampling nucleotides from the genome) were on average longer than all disease-associated genes, further indicating that gene length was a strong driver of mutability (dashed horizontal line, Figure 1B).

A sensitivity analysis stratifying NDD genes by sequence length quartiles demonstrated a strong role of sequence length in NDD gene mutability (Supplementary Figure S2). Genes in the top quartile had an average length of 678,000 nucleotides and were mutated more than the expected per-gene mutation rate by all chemical exposure classes. By contrast, the bottom quartile genes averaged 20,000 nucleotides in length and were mutated less than the expected mutation rate by several exposures (Supplementary Figure S2).

Another sensitivity analysis explored the mutability of cancer driver genes, and genes overlapping between cancer and neurodevelopmental processes (Supplementary Figure S3). NDD genes were mutated at higher-than-expected rates by all chemical classes except for nitrosamines, while cancer driver genes were never mutated more than expected. Furthermore, genes overlapping between the NDD and cancer lists were never mutated more than expected.

### Gene ontology

Gene ontology analysis on the 692 genes which contained coding sequence (CDS) variants in PAH-treated iPSCs revealed enriched gene ontologies closely related to neurodevelopment: neuron projection, neuron part, and calcium ion binding (Supplementary Table S4). Furthermore, the enriched plasma membrane term may be related to metabolic diseases including obesity and type 2 diabetes (Cheng et al., 2018). Similar results were obtained when the analysis included all 2,061 CDS mutations in iPSCs exposed to all environmental mutagens rather than just PAH-treated iPSCs. For instance, the top three gene ontology terms were neuron projection guidance, sensory organ morphogenesis, and cell morphogenesis involved in neuron differentiation (Supplementary Table S5), supporting our hypothesis that NDD genes are particularly vulnerable to environmental mutagens.

In addition to the vulnerability of NDD genes to environmental mutagens uncovered here, genes associated with obesity and type 2 diabetes were mutated by chemical treatment more than expected. We therefore hypothesized that these genes might be linked to neurodevelopmental processes. To explore this possibility, we performed GO analysis on our list of obesity- and type 2 diabetes-associated genes, but found no evidence for enrichment of NDD processes (Supplementary Table S6).

### Sequence characteristics and mutation vulnerability

**Figure 4:**
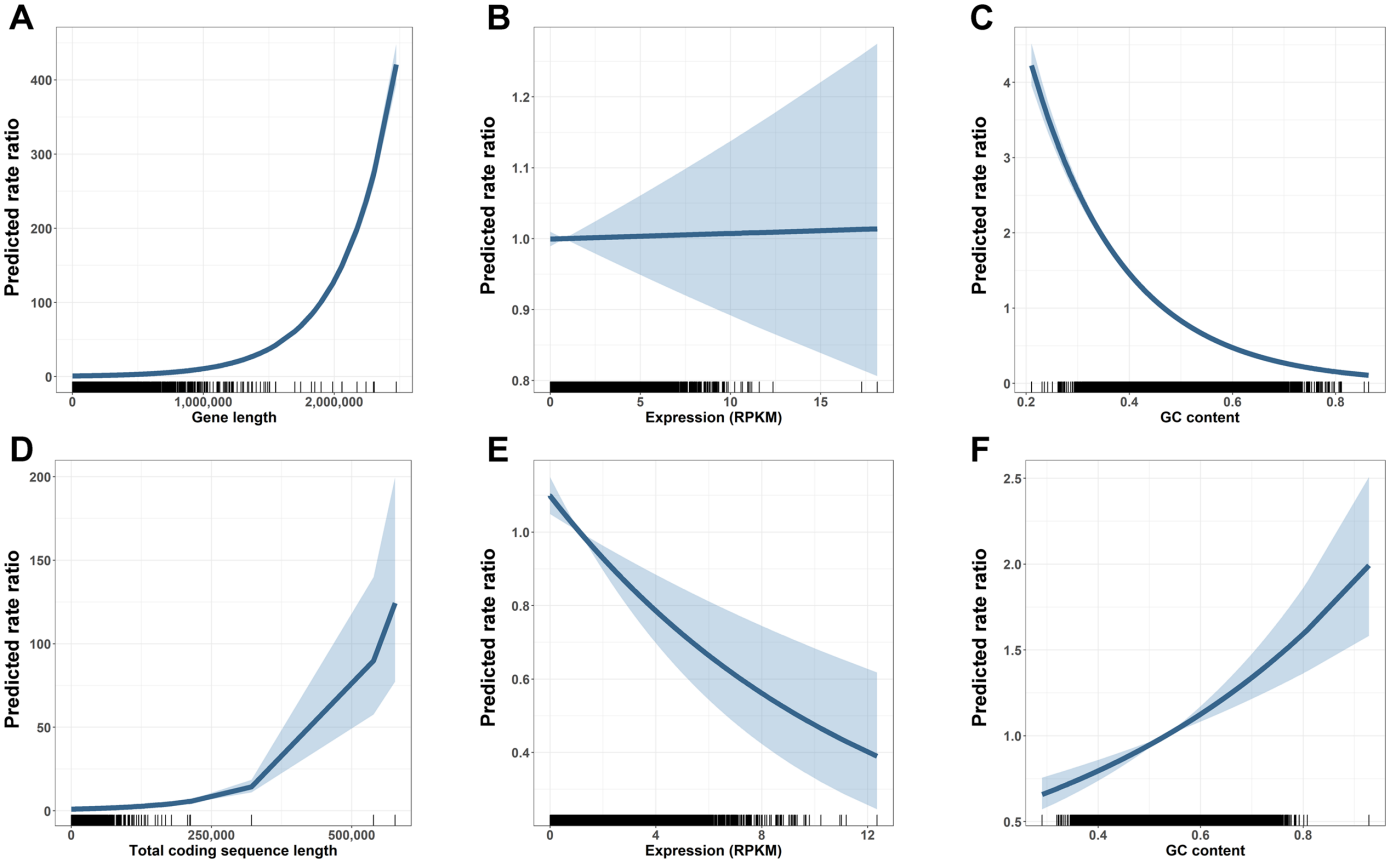
Gene length and other determinants of mutation vulnerability across gene bodies and coding sequences. Gene body Quasi-Poisson model results **(A–C)** depict centered rate ratios (lines) and 95% confidence intervals (shaded regions) at each value of *x* (i.e., panel A shows the predicted mutation rate ratio of a gene with gene length indicated on the *x*-axis versus a gene with length, expression, and GC content set to the mean). Distribution of gene characteristics shown along the x axis, with one mark per observation. Longer gene length is associated with higher mutation frequencies across carcinogen treated cells **(A)**. Expression is not associated with altered mutation risk across gene bodies **(B)**. Higher GC content is associated with decreased mutation risk across gene bodies **(C)**. Coding sequence (CDS) Quasi-Poisson model results **(D–F)** depict centered rate ratios (lines) and 95% confidence intervals (shaded regions) at each value of *x*. Distribution of CDS characteristics shown along the *x* axis, with one mark per observation. Longer coding sequence is associated with a modestly increased risk of mutation **(D)**. Higher gene expression is associated with reduced CDS mutations **(E)**. In contrast to the gene body, higher GC content is associated with increased risk of CDS mutation **(F)**.

Quasi-Poisson regressions further supported a stronger role of sequence length in gene but not CDS mutation number. Controlling for expression and GC content, each interquartile range increase (IQR) in gene length was associated with a 1.104-fold increase in gene mutation rate (rate ratio (RR) = 1.104, 95% CI [1.102, 1.105]; Figure 4A), while an IQR-increase in CDS length was associated with a 1.050-fold increased CDS mutation rate (RR = 1.050, 95% CI [1.045, 1.055]; Figure 4D).

Quasi-Poisson regressions also showed significant effects of GC content and expression on gene and CDS mutability. Expression was not associated with mutability for genes (RR = 1.001, 95% CI [0.988, 1.014]; Figure 4B), while each IQR increase in expression was associated with a 13% decreased mutation rate for CDS (RR = 0.870, 95% CI [0.812, 0.931]; Figure 4E). Similarly, the consequence of GC content was different for genes and CDS. Each IQR-increase in gene GC content was associated with a 0.524-fold decreased mutation rate (RR = 0.524, 95% CI [0.508, 0.539]; Figure 4C), while each IQR-increase in CDS GC content was associated with a 1.274-fold increased mutation rate (RR = 1.274, 95% CI [1.175, 1.381]; Figure 4F).

### Local sequence and mutation vulnerability

**Figure 5:**
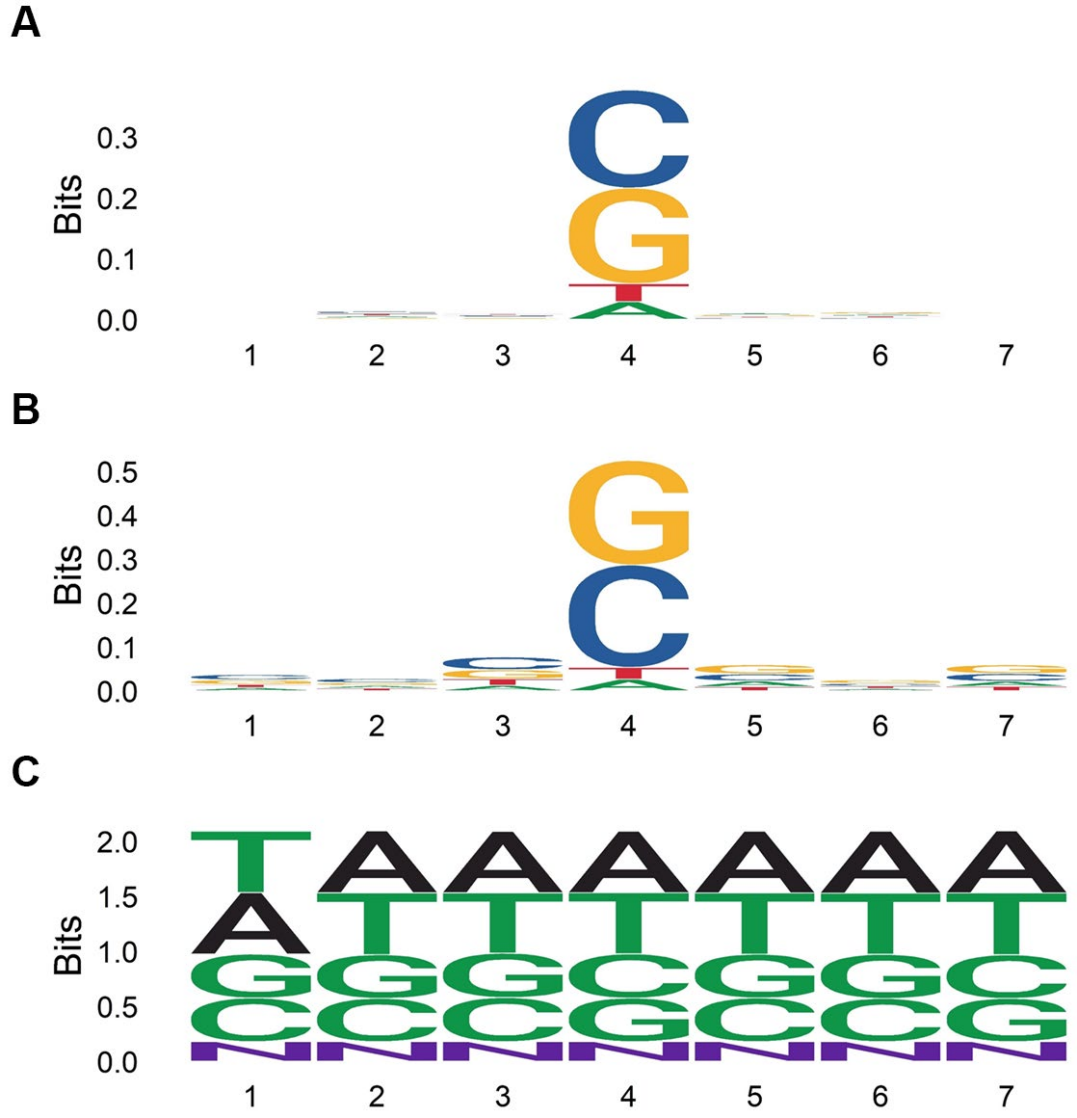
Nucleotide content of 7-mers centered on each gene body **(A)** or coding sequence **(B)** mutation among all single base substitutions identified by Kucab et al. (2019), and for 50,000 7-mers randomly sampled from the human genome **(C)**.

**Figure 6:**
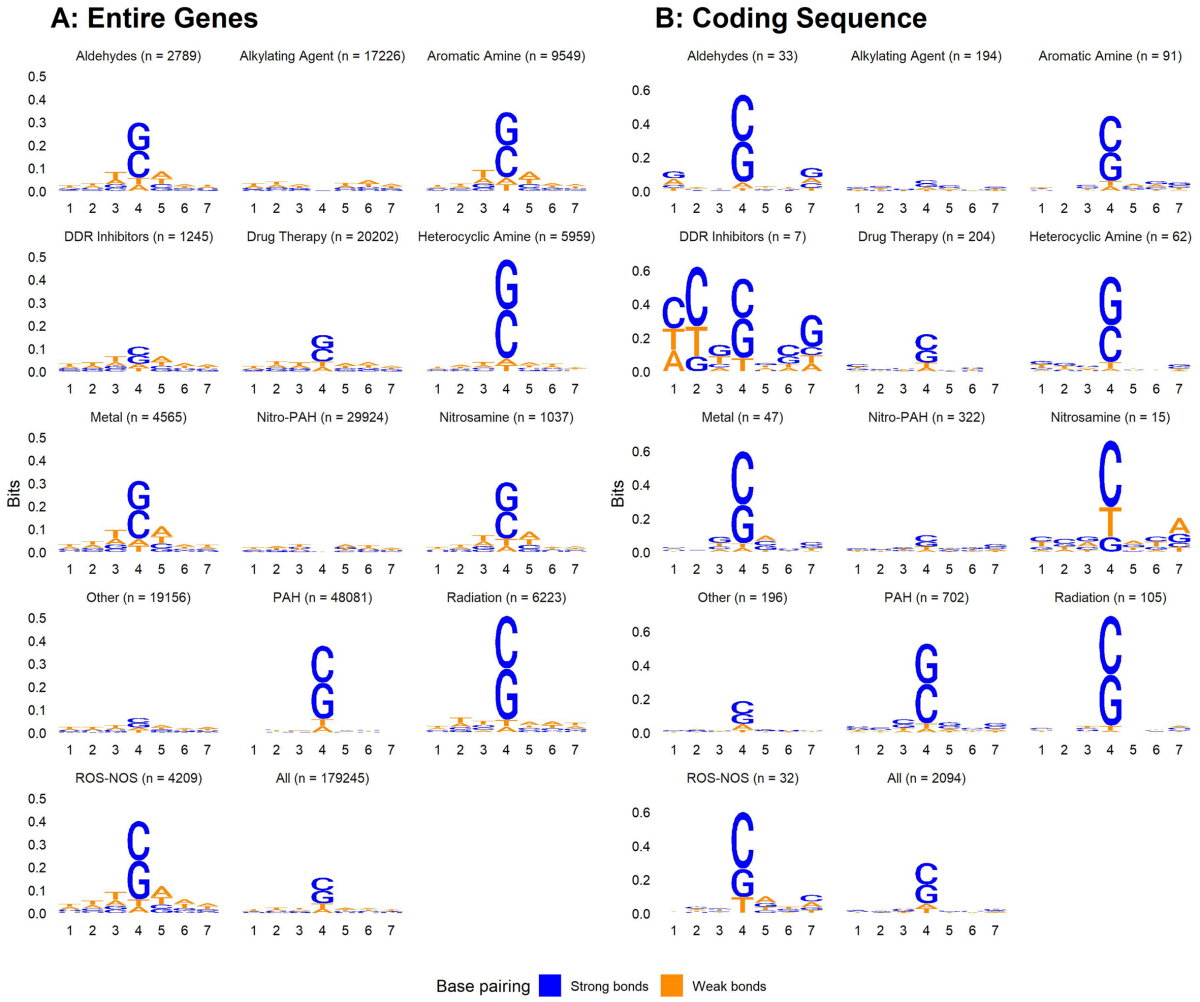
Nucleotide content of 7-mers centered on each gene body **(A)** or coding sequence **(B)** mutation among all single base substitutions identified by Kucab et al. (2019), stratified by chemical class.

We aligned 7-mers centered on each gene or CDS mutation, along with randomly sampled 7-mers from the human genome (Figure 5). Mutated regions were GC enriched, while randomly sampled 7-mers contained equal proportions of each nucleotide. G and C were more highly enriched in 7-mers centered on CDS mutations compared to 7-mers centered on gene mutations. Thus, CDS mutations may be governed more strongly by local GC content. When aligning 7-mers on each gene or CDS mutation stratified by chemical exposure, this pattern held for some but not all chemical classes (Figure 6).

**Figure 7:**
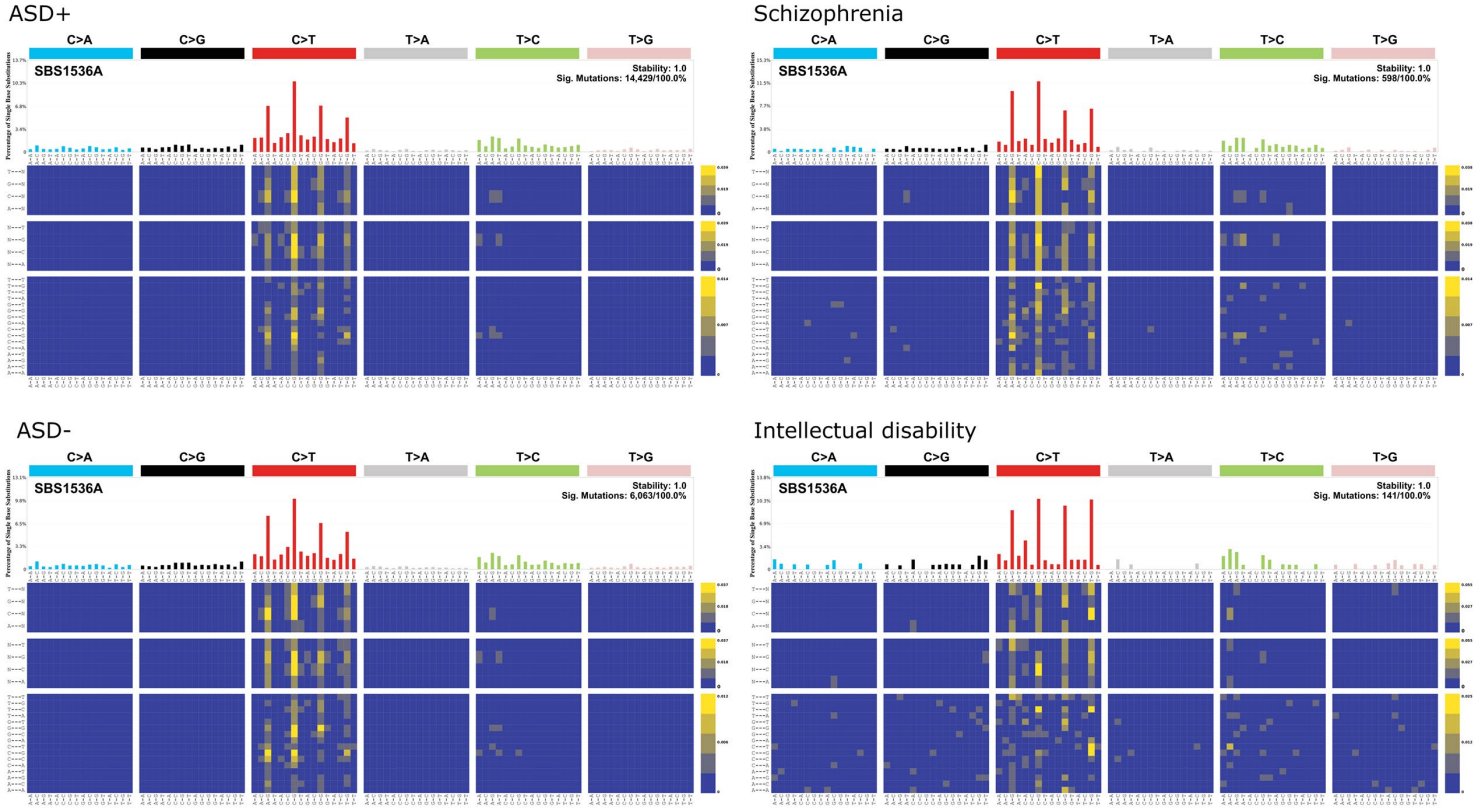
COSMIC single base substitution signatures for *de novo* mutations in individuals living with autism spectrum disorders (ASD) and their family members as controls (ASD–; Feliciano et al., 2018), schizophrenia (Fromer et al., 2014), and intellectual disability (De Ligt et al., 2012).

In the COSMIC mutational signatures analysis, single base substitution enrichments for all neuropsychiatric cases and controls were clock-like/aging associated signatures (i.e., SBS1; Figure 7), which are enriched for NpCpG to NpTpG substitutions. The SBS1 signature does not resemble any of the chemical mutation signatures identified by Kucab et al. (2019). One could interpret this preliminary analysis to suggest that *de novo* mutations in ASD, schizophrenia, and intellectual disability reflect sporadic mutational processes rather than chemical-induced mutation. However, it is also possible that mutational signatures generated from iPSC cultures are not readily comparable to *in vivo* human mutational signatures. For instance, methylated CpG sequences are disproportionately targeted by environmental carcinogens such as PAHs, which form guanine adducts that induce G to T transversions at methylated CpGs (Pfeifer, 2006).

### Polycyclic aromatic hydrocarbon adduct repair associated mutation

**Figure 8:**
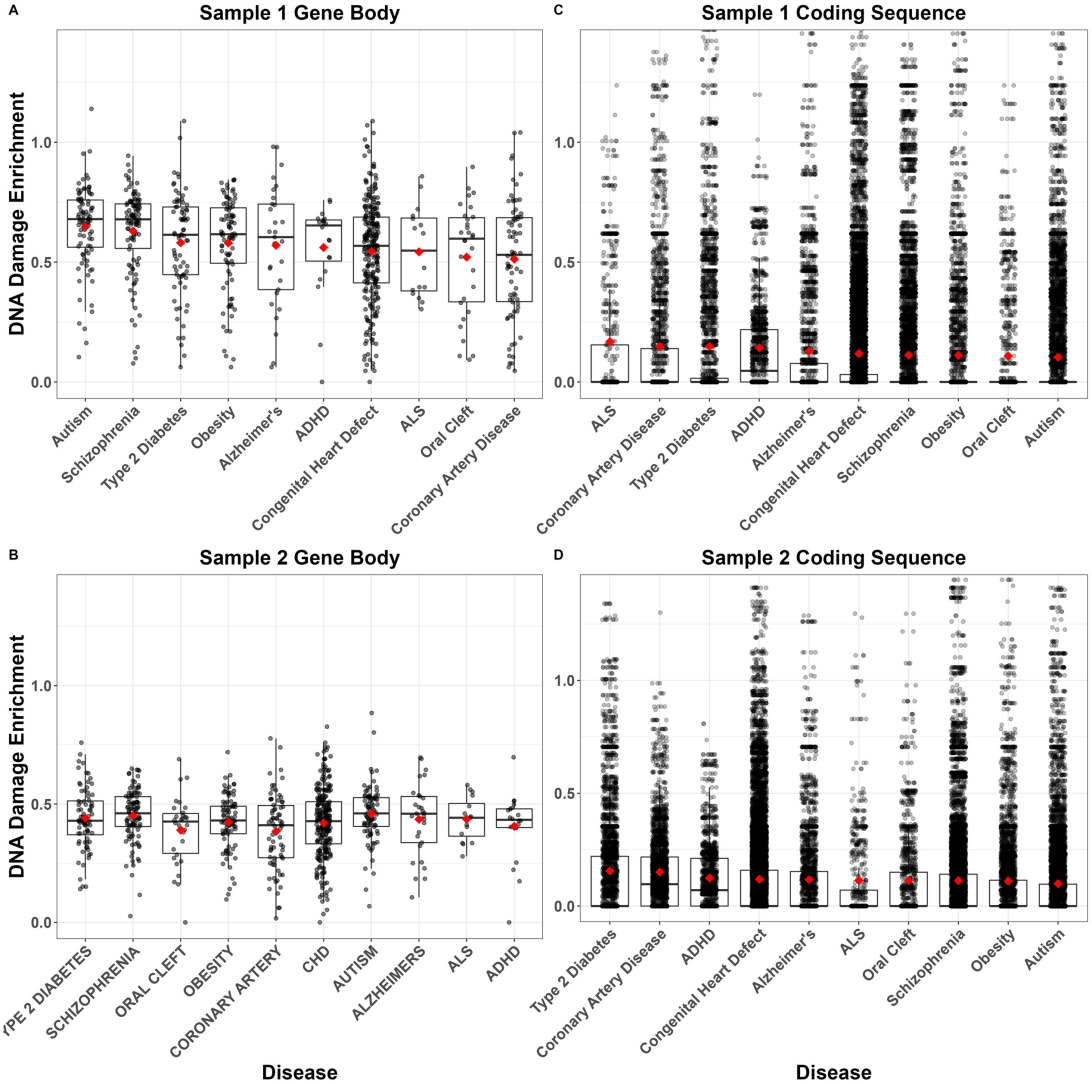
Average DNA damage enrichment following polycyclic aromatic hydrocarbon (PAH) treatment of GM12878 cells across each gene body **(A,B)** or coding sequence **(C,D)** in each of 10 disease-related gene sets. Enrichment values indicate the number of tXR-seq counts per gene/coding sequence, controlling for sequence length. Boxplots show median and interquartile range (IQR) with 1.5 IQR whiskers. Gene sets are ordered left–right within each panel from highest to lowest mean enrichment (red diamonds). Data come from two samples of treated cells from Li et al. (2017).

An existing genome-wide PAH adduct repair assay dataset (Li et al., 2017) was utilized to determine if NDD genes are linked to PAH adduct repair. Pairwise contrasts revealed that DNA damage following PAH treatment was significantly enriched in genes of ASD-related genes compared to genes associated with coronary artery disease, congenital heart defects, and orofacial cleft (Figures 8A, B; Supplementary Table S7). Schizophrenia genes similarly demonstrated more DNA damage compared to coronary artery disease and congenital heart defect genes (Figures 8A, B). However, this pattern of increased DNA damage in neurodevelopmental diseases was not observed for CDS. In fact, ASD and schizophrenia CDS were among the diseases with the lowest levels of DNA damage (Figures 8C, D). Although over half of the Dunn’s Tests contrasts were significant (Supplementary Table S7), differences in the mean and median CDS DNA damage enrichments between diseases were minimal (Figures 8C, D).

## Discussion

We have shown that environmental carcinogens may disproportionately mutate neurodevelopmental and metabolic genes. ASD, ADHD, schizophrenia, obesity, and type-2 diabetes genes were mutated significantly more than expected based on the mutation rate of randomly sampled genes, while GO analyses revealed that genes mutated by PAHs and other environmental carcinogens were overwhelmingly enriched for neurodevelopmental processes. Environmentally induced mutations may play a greater role in neurodevelopmental disease than previously assumed. Rather than attributing sporadic neurodevelopmental diseases to intrinsic mutational processes, this work suggests that some proportion of genetic neurodevelopmental disease risk may be explained by environmental mutagenesis.

Neurodevelopmental genes may be particularly sensitive to mutation because the transcriptome of neural tissues, especially neurons, is biased toward longer genes (King et al., 2013; Zylka et al., 2015). Our analyses revealed that sequence length was a strong driver of mutability, although the association between sequence length and mutability was twice as strong for genes compared to CDS. Other factors may more strongly govern the mutability of protein coding sequences. For instance, we found that higher expression was associated with lower CDS mutation rate, while expression had no effect on the mutability of entire genes. This corroborates prior work showing that lowly expressed genes harbor more mutations (Pleasance et al., 2010), a phenomenon that might be attributable to transcription-coupled DNA repair (Fousteri and Mullenders, 2008; Hanawalt and Spivak, 2008).

Similarly, the effect of GC content was different for genes and CDS. Increased GC content was associated with fewer mutations in genes, but more mutations in CDS. These results are consistent with prior studies indicating that the effect of GC content on mutation rate varies over different genomic scales. GC content across entire genes may reflect higher order DNA structure, and increased GC content has been shown to correlate with decreased mutation rate at higher genomic scales (Wolfe et al., 1989; Hodgkinson and Eyre-Walker, 2011). CDS GC content, however, may more accurately reflect the effect of local GC content on mutability. Cytosines may experience higher mutation rates than other bases because methylated cytosines in CpG dinucleotides are vulnerable to deamination into thymidine. Furthermore, these mutations occur at higher rates in regions with higher local GC content (Fryxell and Moon, 2005).

Our analyses of the relationship between gene characteristics and mutability show that sequence length is a strong driver, but not the only factor contributing to the elevated mutability of neurodevelopmental disease genes in this dataset. However, our models excluded several characteristics known to be associated with mutation. Studies of cancer driver genes have more comprehensively examined associations of gene characteristics with mutability (e.g., Lawrence et al., 2013; Gorlov et al., 2018). Additional gene and/or sequence characteristics examined in relation to mutability include open versus closed chromatin state (Ying et al., 2010; Schuster-Böckler and Lehner, 2012; Thurman et al., 2012), epigenetic markers (Coarfa et al., 2014), replication timing (earlier replicating regions have a lower mutation rate: Lang and Murray, 2011), di- and/or tri-nucleotide composition (Millar et al., 2002; Samocha et al., 2014), evolutionary conservation (Michaelson et al., 2012), and protein-DNA interactions identified *via* ChIP-seq (e.g., transcription factor binding) (Yang et al., 2018).

Although *de novo* mutation has previously been hypothesized as a pathway linking environmental exposures to increased NDD risk, particularly ASD (Kinney et al., 2010; Pugsley et al., 2021), this hypothesis has not been explicitly tested. For instance, epidemiologic research has linked many known carcinogens, such as air pollutants and heavy metals, with elevated ASD rates at the population level, but none of these studies include mutation data [reviewed by Pugsley et al. (2021)]. Addressing this limitation will require formal mediation analyses showing associations of environmental exposures with increased *de novo* mutation rates, which in turn result in elevated incidence of neurodevelopmental disease. We are unaware of any studies employing this type of mediation approach for environmental exposures, although the mediating role of *de novo* mutations has been investigated for paternal age (Gratten et al., 2016; Taylor et al., 2019). In the future, whole genome/exome sequencing studies of neurodevelopmental diseases such as ASD will need to collect data on environmental exposures to assess this hypothesis.

This work has several limitations. First, our findings that environmental chemicals may disproportionately mutate neurodevelopmental disease genes supports but is not an explicit test of the hypothesis described above. Second, the methods of gene set curation could bias our analyses comparing observed rates of exposure-induced mutations in disease gene sets with the mutation rate of control genes randomly sampled from the genome. To partially address this limitation, we created an online tool allowing researchers to query their own gene sets. Another limitation was our reliance on mutations called in cultured human iPSCs rather than *in vivo*. Because PAHs are a major air pollutant, we attempted to externally validate the results from PAH-exposed iPSCs by analyzing lung tumor mutations from humans living in highly polluted regions. However, future studies might better validate these results using animal models dosed with comparable levels of the environmental chemicals examined by Kucab et al. (2019). Additionally, the use of one iPSC line precludes an examination of potential genetic variability in gene-set mutation vulnerability. Future research should account for diverse genetic backgrounds in genomic instability in disease specific *de novo* mutations.

## Author’s note

Custom gene lists can be queried using the algorithm we generated at www.environmentalmutation.com.

## Data availability statement

Publicly available datasets were analyzed in this study. This data can be found here: Data are available from the primary sources cited. The SPARK gene variants are available to approved researchers through SFARI upon review. The code generated during this study are available on GitHub at: https://github.com/brennanhilton/environmental-mutation-calculator.

## Author contributions

BB, JS, WC, and BP contributed to conception and design of the study. BB and SZ performed the statistical analysis. BB wrote the first draft of the manuscript. All authors contributed to manuscript revision, read, and approved the submitted version.

## Funding

This work was supported by the NIH grants: P30ES009089, R21ES032913, and R24ES029489.

## Conflict of interest

The authors declare that the research was conducted in the absence of any commercial or financial relationships that could be construed as a potential conflict of interest.

## Publisher’s note

All claims expressed in this article are solely those of the authors and do not necessarily represent those of their affiliated organizations, or those of the publisher, the editors and the reviewers. Any product that may be evaluated in this article, or claim that may be made by its manufacturer, is not guaranteed or endorsed by the publisher.

## Footnotes

^1^http://environmentalmutation.com

^2^https://www.gencodegenes.org/human/
