# Supplementary material for: Environmental carcinogens disproportionally mutate genes implicated in neurodevelopmental disorders
Source: Front Neurosci. 2023 Aug 3;17:1106573. doi: 10.3389/fnins.2023.1106573 (PMC10435087; doi:10.3389/fnins.2023.1106573)
Supplement: Supplementary file 2 [file Data_Sheet_2.docx]

Supplemental Figure 1


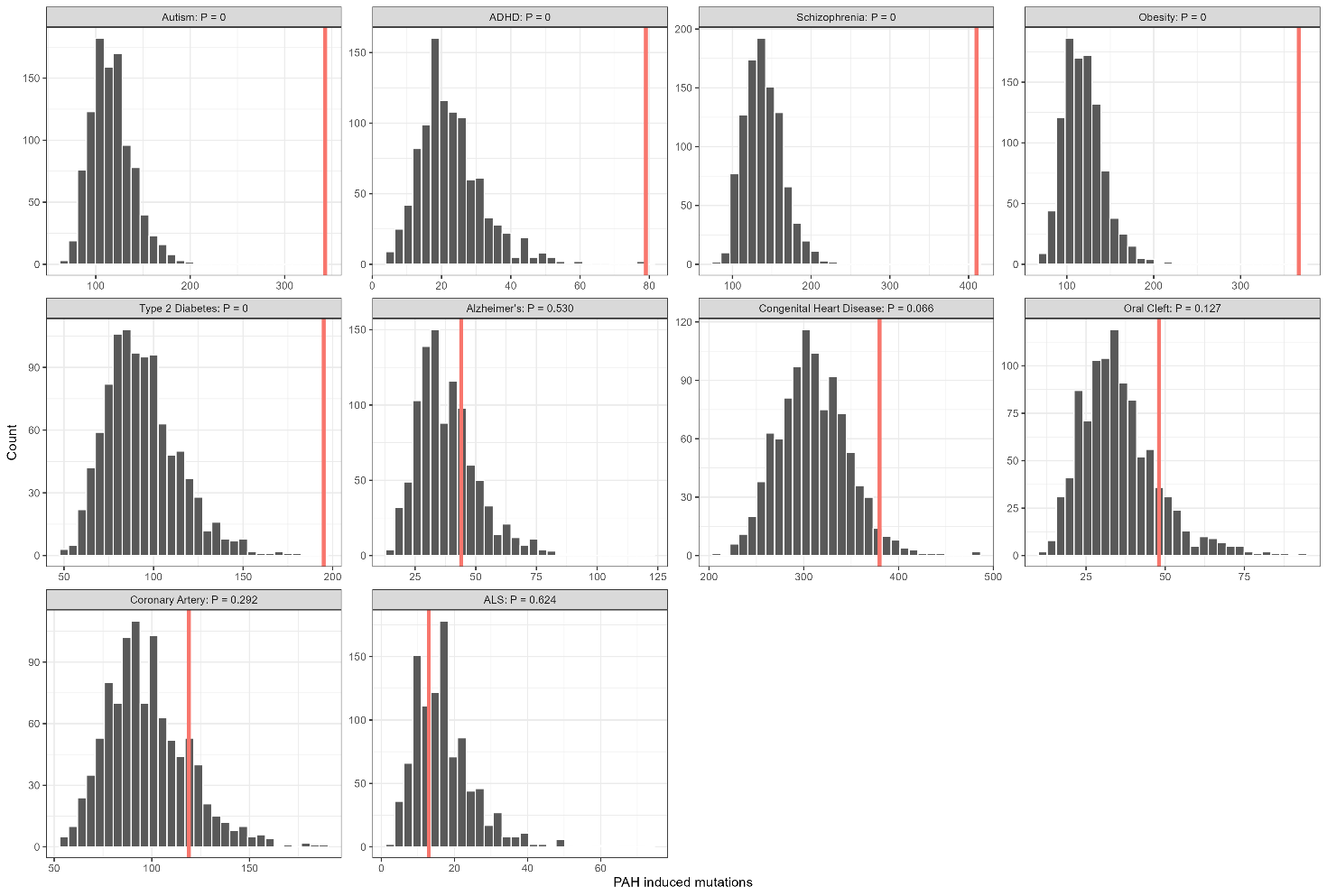


Legend: Monte Carlo null distributions for each disease gene set were obtained by randomly sampling 1,000 sets of genes from the human genome equal to the number of genes in a given disease gene set. The number of mutations in randomly sampled genes in PAH-treated iPSCs were determined and plotted as histograms (black bars). Red lines indicate the number of mutations in each disease gene set. Two-tailed P-values were calculated as the proportion of randomly sampled gene sets mutated more or less than the disease gene set, whichever was smallest, multiplied by two.

Supplemental Figure 2


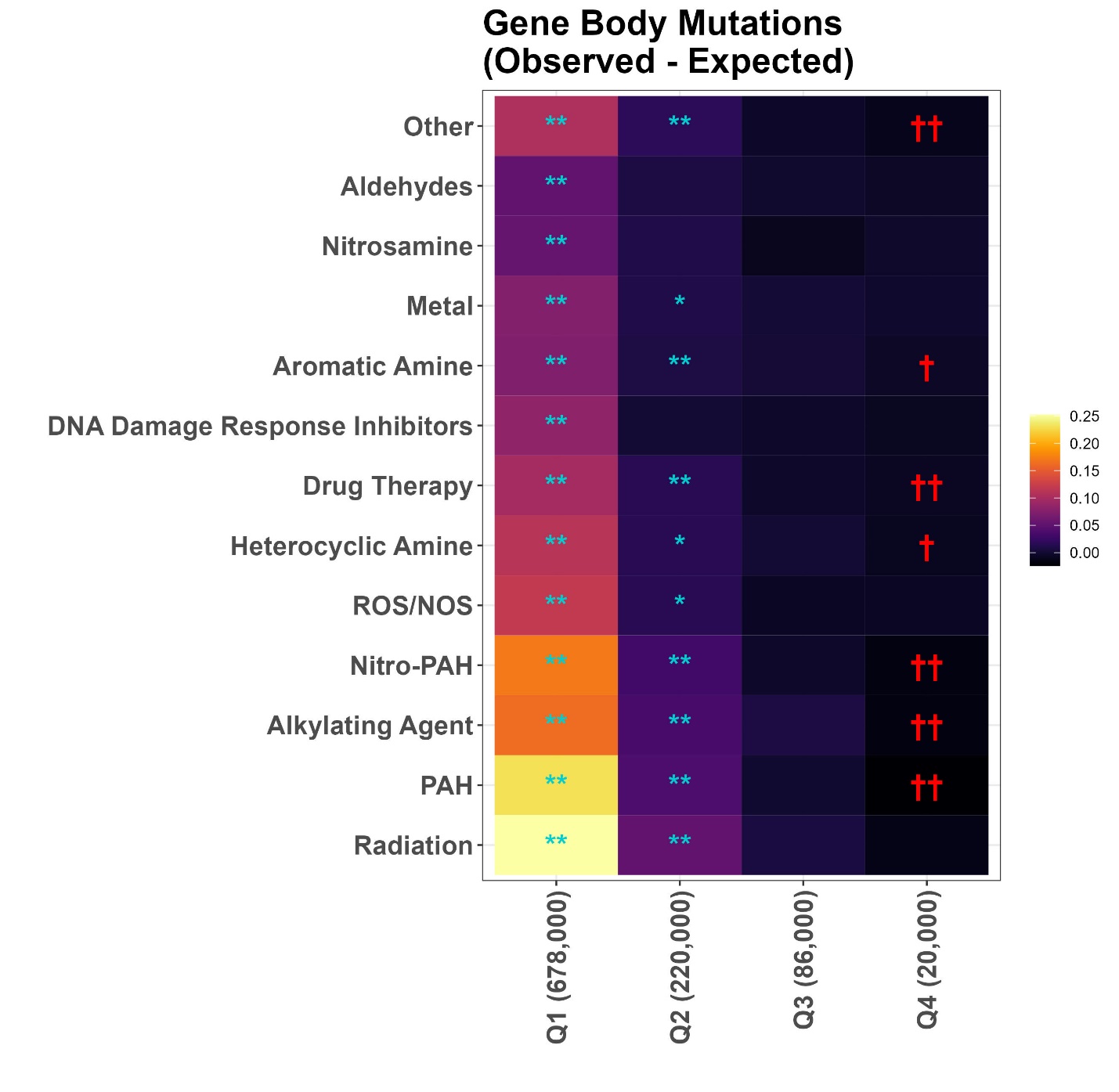


Legend: Mutation vulnerability of disease associated genes to various carcinogens. Heatmaps of observed minus expected mutations in disease gene sets for mutations in gene body sequence following chemical treatment in iPSC. Gene sets indicated on x-axis are NDD genes divided into sequence length quartiles (Q1-Q4) with mean sequence length rounded to the nearest 1,000 specified. NDD gene set comes from combination of ASD, ADHD, and schizophrenia genes. Significant levels from exact binomial tests. Higher mutation rates: * p<.05; ** Bonferroni-adjusted p<.05. Lower mutation rates: † p<.05; †† Bonferroni-adjusted p<.05.

Supplemental Figure 3


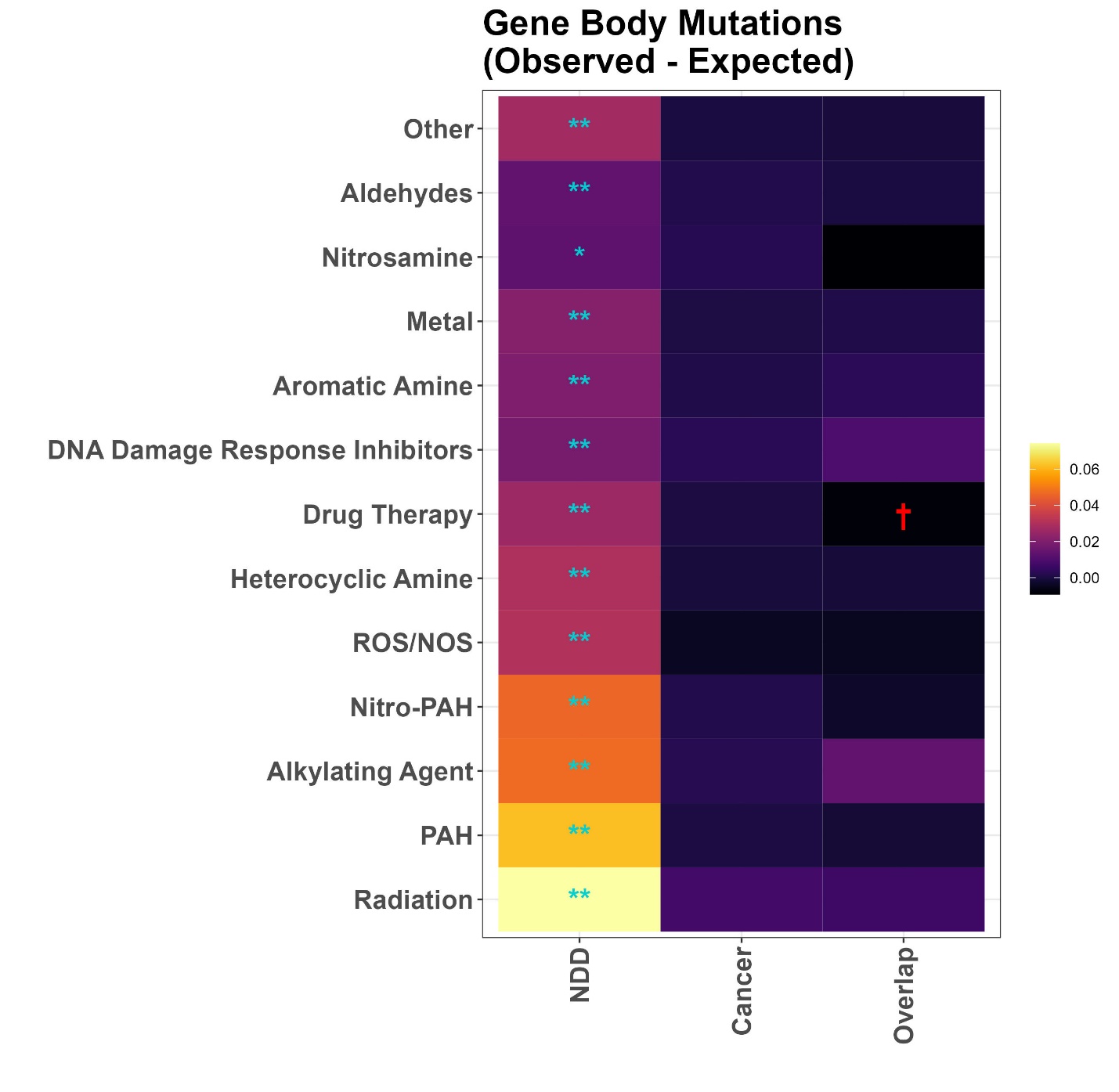


Legend: Mutation vulnerability of disease associated genes to various carcinogens. Heatmaps of observed minus expected mutations in disease gene sets for mutations in gene body sequence following chemical treatment in iPSC. Gene set indicated on x-axis. NDD gene set comes from combination of ASD, ADHD, and schizophrenia genes, cancer gene set includes 233 high confidence cancer driver genes, and overlap list contains 14 genes found in both the NDD and cancer gene sets (see methods). Significant levels from exact binomial tests. Higher mutation rates: * p<.05; ** Bonferroni-adjusted p<.05. Lower mutation rates: † p<.05; †† Bonferroni-adjusted p<.05.
